# Autonomic symptoms in migraine: Results of a prospective longitudinal study

**DOI:** 10.3389/fneur.2022.1036798

**Published:** 2022-11-03

**Authors:** Jason C. Ray, Sanjay Cheema, Emma Foster, Lakshini Gunasekera, Dwij Mehta, Susan J. Corcoran, Manjit S. Matharu, Elspeth J. Hutton

**Affiliations:** ^1^Department of Neurology, Alfred Hospital, Melbourne, VIC, Australia; ^2^Department of Neuroscience, Monash University, Melbourne, VIC, Australia; ^3^Department of Neurology, Austin Health, Heidelberg, VIC, Australia; ^4^Headache and Facial Pain Group, University College London (UCL) Queen Square Institute of Neurology, The National Hospital for Neurology and Neurosurgery, London, United Kingdom; ^5^Department of Neurology, Barwon Health, Geelong, VIC, Australia; ^6^Department of Cardiology, Alfred Hospital, Melbourne, VIC, Australia

**Keywords:** migraine, autonomic, COMPASS-31, dysautonomia, autonomic (vegetative) nervous system

## Abstract

**Objective:**

To assess the prevalence and burden of autonomic symptoms in migraine, and determine the relationship with migraine frequency.

**Background:**

Autonomic symptoms in migraine have been theorized to occur in the setting of inter-ictal sympathetic hypoactivity and hyper-sensitivity. There is limited data prospectively assessing cranial and extra-cranial autonomic symptoms with a validated instrument, or longitudinal data on the relationship between migraine disease activity and autonomic symptoms.

**Methods:**

Patients attending a single tertiary academic center were recruited into a prospective cohort study between September 2020 and June 2022. In addition to standard clinical care, they completed several surveys including the Composite Autonomic Symptom Scale (COMPASS-31) questionnaire, a validated survey of autonomic symptoms.

**Results:**

A total of 43 patients (66.7% female, median age 42, IQR 17) were included in the final analysis. There was a baseline 20 monthly headache days (MHD) (IQR 21.7), and 65.1% of the population had chronic migraine by ICHD-3 criteria. A significantly elevated weighted COMPASS-31 score was reported in 60.5% of respondents (mean 30.3, SD 13.3) at baseline. After 12 months treatment, significant improvements were reported in migraine frequency (median MHD 20–8.7) and disability (median Migraine Disability Assessment Score 67–48), but not in autonomic symptoms (mean score 30.3, SD 11.2).

**Conclusion:**

Autonomic symptoms were frequently reported in patients with migraine. However, they did not correlate with headache frequency or reversion to episodic frequency. Further study is required to elucidate specific approaches and treatments for autonomic symptoms, and further evaluate the underlying pathophysiological mechanisms.

## Introduction

Autonomic symptoms are commonly experienced by people living with migraine, both ictally and inter-ictally. The International Classification of Headache Disorders, third edition (ICHD-3) describes key cranial autonomic symptoms in headache disorders; conjunctival injection, lacrimation, nasal congestion, rhinorrhoea, eyelid oedema, forehead/facial sweating, forehead and facial flushing, aural fullness, miosis and ptosis (1). Approximately 40–50% of patients with migraine report at least one cranial autonomic symptom during ictus (2, 3).

The presence of extra-cranial autonomic symptoms in migraine have increasingly been recognized. The population-based CAMERA study identified that patients with migraine have higher rates of orthostatic intolerance (32 vs. 12%), and frequent syncopal episodes (13 vs. 5%) compared to the general population, irrespective of migraine disease frequency or severity (4). Well-described extra-cranial autonomic symptoms in migraine include orthostatic intolerance, nausea, diarrhea and polyuria (5).

Given the burden of autonomic symptoms in migraine, studies have previously attempted to assess sympathetic and parasympathetic function in migraine. This literature has been ably summarized elsewhere (6, 7). The majority, but not all, of the studies suggest a trend to sympathetic hypoactivity inter-ictally, and an exaggerated sympathetic response ictally (7). One hypothesis for this observation is that repeated ictal stimulation of the sympathetic autonomic system leads to downregulation and reduced plasma norepinephrine release, which over time leads to upregulation of postsynaptic adrenergic receptors and the resultant ictal hypersensitivity (6). This theory correlates with previous observations that the presence of cranial autonomic symptoms have been found to occur more frequently in patients with central sensitization, compared to those without (2).

Despite the prevalence and significance of autonomic symptoms in migraine, there remains a paucity of data prospectively assessing for autonomic symptoms in patients with migraine, or the relationship of autonomic symptoms with headache disability. The goals of this study were to prospectively assess autonomic symptoms in migraine, and evaluate the relationship of autonomic symptoms with migraine disease state and severity.

## Methodology

### Study design

This was a prospective, longitudinal cohort study with participants actively recruited from a major metropolitan hospital's outpatient headache clinic between September 2020 and June 2022.

### Research participants

Participants meeting ICHD-3 diagnostic criteria for episodic or chronic migraine (1), as assessed by headache specialists, were enrolled from the Headache Clinic of the Alfred Hospital, Melbourne, Australia.

In addition to standard clinical care, participants were invited to complete several clinical surveys. Migraine disability was captured utilizing the Migraine Disability Assessment Score (MIDAS). The minimally important difference of MIDAS has been calculated as a score of 5 (8). Patients also completed the Beck Depression Inventory (BDI) and the General Anxiety Disorder (GAD-7) surveys. Autonomic symptoms were sampled utilizing the Composite Autonomic Symptom Scale (COMPASS-31) questionnaire.

The COMPASS-31 questionnaire (Appendix 1) is a validated survey based on the Autonomic Symptom Profile. The survey asks 31 questions, providing a weighted score in six sub-domains of symptoms; orthostatic intolerance (range 0–40), vasomotor (range 0–5), secretomotor (range 0–15), gastrointestinal (range 0–25), bladder (range 0–10) and pupillomotor (range 0–5). An overall score totals these sub-domains, with a maximum possible score of 100 indicating severe autonomic symptoms (9–11). It has been shown to have internal validity and test-retest reliability (12). Participants were invited to complete the surveys at baseline and after 12 months. Patient results were calculated from the longest follow-up period.

### Statistical considerations

Statistical analysis was performed using SPSS v28.0. Test results were considered significant when *p* < 0.05. Sample size was determined on the basis of group differences from previously published literature (13). A sample size of 26 was required to provide 95% confidence of providing a 0.67 effect size of difference between groups. Tests of normality were determined using the Shapiro-Wilk (SW) test. COMPASS-31 score was normally distributed (SW = 0.989, *p* = 0.957), non-normally distributed data included monthly headache days (MHD) (SW = 0.873, *p* < 0.001), MIDAS (SW = 0.776, *p* < 0.001), BDI (SW = 0.917, *p* = 0.004), and GAD-7 (SW = 0.940, *p* = 0.027).

Population characteristics were summarized with descriptive statistics. Longitudinal change was assessed with paired samples *T*-test for normally distributed data, and Wilcoxon signed rank test for non-normally distributed data. An exploratory analysis was undertaken to evaluate the correlation of autonomic symptoms with headache frequency, depression, anxiety, or change in disease state. A Spearman rank correlation coefficient was computed to assess the relationship between baseline MHD frequency, MIDAS score and COMPASS-31 score. There was no correction undertaken for multiple comparisons. This study has received institutional review board approval (HREC 217/20).

## Results

Over the study period, 105 patients were assessed for eligibility for the study. Following review, 26 patients were excluded. Four patients were subsequently diagnosed with cluster headache, and 22 patients had comorbid medical conditions that are also associated with autonomic symptoms, i.e., multiple sclerosis and postural tachycardia syndrome. A total of 79 patients were enrolled, and 43 patients completed 12 months of treatment as well as follow-up surveys, and were included in the final analysis. At baseline, median MHD were 20 (Interquartile range, IQR 21.7), and median MIDAS of 67 (standard deviation, SD 154), indicative of a severe level of disability. A COMPASS-31 score ≥30 was reported in 26/43 (60.5%) of participants. Characteristics of the patient population are provided in Table 1.

**Table 1:** Population characteristics.

	**Baseline cohort**	**Follow-up**	
	***N* = 43**	***N* = 43**	
Age Median (IQR)	42 (17)		
Female *n* (%)	30 (66.7)		
CM *n* (%)	28 (65.1)	16 (37.2)	*p* = 0.002
MHD Median (IQR)	20 (21.7)	8.7 (22.7)	*p* = 0.001
MIDAS Median (IQR)	67 (154)	48 (65)	*p* = 0.008
BDI Median (IQR)	15 (15)	13 (14)	*p* = 0.309
GAD-7 Median (IQR)	7 (8)	2 (6)	*p* < 0.001

CM, chronic migraine; MHD, monthly headache days; MIDAS, migraine disability assessment test. BDI, beck's depression inventory; GAD-7, generalized anxiety disorder 7-item assessment.

**Figure 1:**
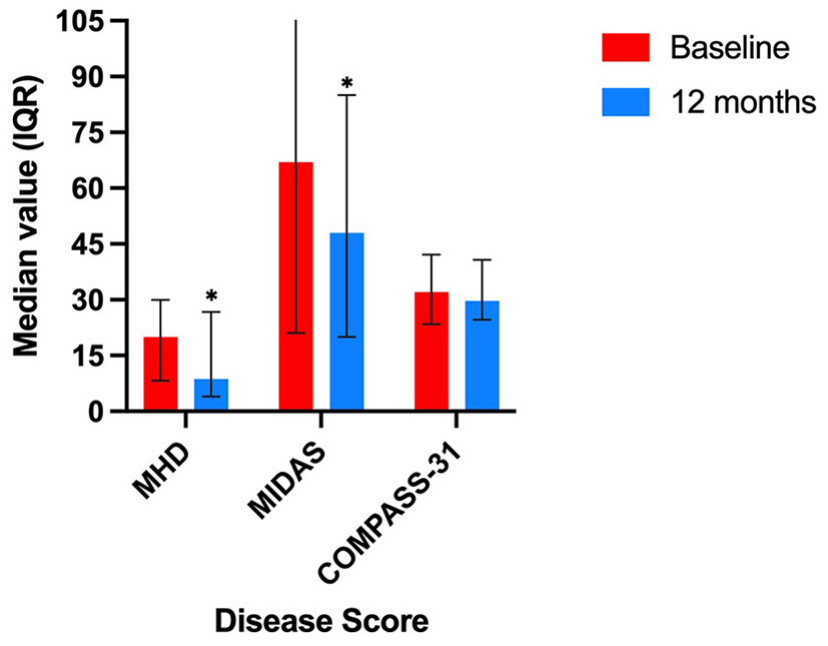
Response of cohort by symptom score over 12 month follow-up period. MHD, median monthly headache days; MIDAS, median Migraine Disability Assessment Score; COMPASS-31, median Composite Autonomic Symptom Score, *denotes statistically significant change.

Over the follow-up period, the median MHD significantly reduced from 20.0 to 8.7 days/month (*p* = 0.002, z = −3.2). There was a corresponding significant reduction in patient reported headache disability, with the median MIDAS score reducing from 67 to 48 (*p* = 0.01, z = −2.6). The mean composite weighted COMPASS-31 score in this cohort was 30.3 (SD 13.3), and 30.3 (SD 11.2) after 12 months (Figure 1). There was no significant change in mean weighted COMPASS-31 score over the observation period (*p* = 0.885), or in individual domain scores (Table 2).

**Table 2:** COMPASS-31 autonomic symptom score.

	**Baseline cohort** ***N* = 43** **Mean (SD)**	**Follow-up** ***N* = 43** **Mean (SD)**	
Total weighted score (possible score 0–100)	30.3 (13.3)	30.3 (11.2)	*p* = 0.885
Gastrointestinal (possible score 0–25)	6.6 (3.7)	6.8 (3.5)	*p* = 0.692
Vasomotor (possible score 0–5)	1.0 (1.6)	0.7 (1.4)	*p* = 0.155
Secretomotor (possible score 0–15)	3.6 (3.9)	3.8 (3.7)	*p* = 0.713
Orthostatic Intolerance (possible score 0–40)	18.2 (8.9)	17.9 (8.5)	*p* = 0.806
Bladder (possible score 0–10)	0.8 (1.3)	0.8 (1.3)	*p* = 0.774
Pupillomotor (possible score 0–5)	3.0 (5.0)	2.6 (2.2)	*p* = 0.465

The association between autonomic symptoms and other clinicodemographic factors was explored, and there was no significant correlation between MHD and COMPASS-31 (r = 0.046, *p* = 0.768), or between MIDAS and COMPASS-31 score (r = 0.252, *p* = 0.103). In this cohort, depression and anxiety scores were significantly elevated with a median BDI score at baseline of 15 (IQR 15), and median GAD-7 score of 7 (IQR 8). There was a significant weak positive correlation between BDI and COMPASS-31 score (r = 0.333, *p* = 0.029), and a significant weak positive correlation between GAD-7 and COMPASS-31 score (r = 0.373, *p* = 0.029).

In order to assess the impact of change in migraine disease state on autonomic symptoms, two sub-group analyses were undertaken for patients who reported a clinically meaningful reduction in MIDAS (score reduced by >5 points), and for patients who reverted from chronic to episodic migraine. Neither group had significant reduction in mean COMPASS-31 score. A majority of patients (53.5%, *n* = 23/43) had a clinically meaningful reduction in MIDAS score but did not report a significant reduction in mean COMPASS-31 score (mean −0.9, 95% CI −4.7–2.9, *p* = 0.639). Half of the patients (50%, *n* = 14/28) reverted from chronic to episodic migraine, but also had no significant reduction in COMPASS-31 score (mean −1.1, 95% CI −7.3–5.0, *p* = 0.696). Given that five questions of the COMPASS-31 score (Q27-Q31) in a patient with migraine is likely to be reported in the setting of photophobia, we performed an exploratory analysis excluding these questions from analysis. The mean reported score in this setting remained significantly elevated at baseline (30.3, SD 13.3) and on follow up (30.3, SD 11.2).

## Discussion

This is the first study to utilize the validated COMPASS-31 survey to longitudinally assess autonomic symptoms in a well-characterized cohort of people with migraine. We found that participants reported moderate autonomic symptoms at baseline, and that these did not change over time, despite significant improvement in median monthly headache days over the same time-period. We also found a significant weak correlation between severity of autonomic symptoms and severity of anxiety and depression scores. Our findings provide further insights into the mechanisms of autonomic symptoms in migraine, and may increase clinical awareness of these often debilitating symptoms.

Our findings are concordant with the work of Howard et al., who examined differences in COMPASS-31 scores between participants with migraine and healthy controls in a cross-sectional study (13). Howard et al. reported significantly higher COMPASS-31 scores (mean 27.15, SD 14.37) in participants with migraine, compared to healthy controls (mean 11.67, SD 8.98, *p* = 0.001) (13). In concordance with Howard et al., our study also reports no significant correlation between headache frequency and COMPASS-31 score, and only a weak correlation with MIDAS score (13). Furthermore, we have found no association between change in COMPASS-31 score over time with change in migraine disability or reversion to episodic disease. Given this, it is imperative that clinicians enquire and directly address autonomic symptoms in patients with migraine, as patient symptoms may not improve in concert with headache frequency.

In this study, we describe a weak significant relationship between severity of autonomic symptoms and severity of depression and anxiety scores. While not examined, this relationship may be bidirectional. On one hand autonomic symptoms may exert a substantial health burden on patients, while conversely, patients with concomitant anxiety and depression may often self-report symptomatology. Supporting this theory, increased reporting of somatic symptoms in the setting of depression and anxiety has been reported in other settings (14).

Our findings also provide further insights into the postulated driving mechanism of autonomic symptoms in migraine. The observation that autonomic symptoms do not correlate with baseline headache frequency, or improve in concert with either migraine disability or reversion to episodic disease, suggests that the theory of sensitization of the sympathetic nervous system through repeated stimulation, reduced outflow, and up-regulation of postsynaptic transmitters is an incomplete explanation of ictal and interictal autonomic symptoms in migraine.

A speculative theory on precipitating factors of autonomic symptoms in migraine would include the previously discussed sensitization of the sympathetic nervous system and upregulation of post-synaptic transmitters (6, 7). Contribution from dysregulation of sensory processing pathways is also possible. Disruption of descending inhibitory pathways, required for the perception of nociception in a migraine attack, may be facilitated by excess inhibitory GABA pathways interfacing with noradrenergic pathways in the locus coeruleus, and the parasympathetic reflex arc arising from the superior salivatory nucleus (7, 15–18). Disruption of GABA inhibition in the locus coeruleus and possibly by extension, facilitatory networks in the rostral ventral medulla and periaqueductal gray region, have been hypothesized to result in the facilitation of transmission of sensory information (18). Activation of the periaqueductal gray region, which is also involved in the initiation of the sympathetic autonomic system (19), provides a possible mechanism to explain this activation.

Central sensitization as a result of a failure to engage descending inhibitory nociceptive pathways has been described in other conditions such as fibromyalgia (20). Thus, dysfunction of central common descending networks may facilitate autonomic symptoms in migraine, raising the prospect of an underlying susceptibility to aberrant network function and sensory transmission that exists in patients with migraine, as opposed to solely a mal-adaptive process to repeated stimulation of the sympathetic nervous system. Further study is required to explore the pattern of activation and hypofunction of the sympathetic parasympathetic nervous system in patients with migraine to further explore the etiology of these symptoms.

There are several limitations to this study, which was conducted at a single tertiary center throughout the COVID-19 pandemic. Clinical care was variably disrupted and converted to remote care throughout this time period, which may have impacted on clinical outcomes, and likely contributed to the rate of loss of follow-up. The study population was that of a single academic institution, limiting the generalisability of these findings. We cannot therefore be certain that the study population is representative of the general population. Further, it is possible that the follow-up period was not sufficient to allow reversal of central sensitization in patients who achieved an improvement in their disease state. The persistence of autonomic symptoms and sensitization in migraine has not been previously studied. However, the persistence of cutaneous allodynia, consistent with central sensitization in preclinical models of medication overuse may provide some indication of the expected duration of sensitization following removal of perpetuating stimuli. In these studies, resolution of allodynia was observed within 14 days (21, 22). The COMPASS-31 instrument itself relies on patient reported symptoms, and the weighted score does not differentiate sympathetic from parasympathetic dysfunction. This prevents any conclusions being drawn as to the mechanism or pattern of autonomic system dysfunction. Further work is now required to combine this clinical tool with objective testing of the autonomic nervous system to better identify the pattern of abnormality, and explore propagating factors of autonomic symptoms despite improvements in migraine disease state.

## Conclusion

Autonomic symptoms were frequently reported in this population of patients with migraine, in keeping with previous reports in the literature. Severity of autonomic symptoms do not correlate with headache frequency, response to treatment, or reversion to an episodic frequency within the study follow-up period. Further study is required to confirm these observations in the community, to elucidate specific approaches and treatments for autonomic symptoms, and further evaluate the underlying mechanisms.

## Data availability statement

The raw data supporting the conclusions of this article will be made available by the authors, without undue reservation.

## Ethics statement

The studies involving human participants were reviewed and approved by Alfred Health HREC 217/20. Written informed consent for participation was not required for this study in accordance with the national legislation and the institutional requirements.

## Author contributions

JR and EH developed the concept of the project. JR was responsible for the collection of data and initial analysis and drafting of the primary manuscript. All authors provided extensive review, intellectual input, read, and approved the final manuscript.

## Conflict of interest

Author JR has received funding from the Pharmaceutical Society of Australia and the Limbic supported by unrestricted educational grants from Viatris and Novartis respectively. Author EF has been supported by grants from the NHMRC Medical Postgraduate Research Scholarship APP1150482, The Royal Australasian College of Physicians Research Entry Scholarship, AVANT Doctors In Training Scholarship, and Monash University Bridging Postdoctoral Fellowship, and her institution has also received research grants from Brain Foundation, the Australian Epilepsy Research Fund, the Viertel Foundation, and Lundbeck, outside the submitted work. Author MM serves on the advisory board for Allergan, Novartis, Eli Lilly, Autonomic Technologies Inc., and TEVA and has received payment for the development of educational presentations from Allergan, electroCore, Eli Lilly, Novartis and TEVA. Author EH has served on advisory boards for SanofiGenzyme, Novartis, Teva, Eli Lilly, Allergan, Lundbeck, been involved in clinical trials sponsored by Novartis, Teva, Xalud, Cerecin, and has received payment for educational presentations from Allergan, Teva, Eli Lilly and Novartis. The remaining authors declare that the research was conducted in the absence of any commercial or financial relationships that could be construed as a potential conflict of interest.

## Publisher's note

All claims expressed in this article are solely those of the authors and do not necessarily represent those of their affiliated organizations, or those of the publisher, the editors and the reviewers. Any product that may be evaluated in this article, or claim that may be made by its manufacturer, is not guaranteed or endorsed by the publisher.
