# Supplementary material for: Autonomic symptoms in migraine: Results of a prospective longitudinal study
Source: Front Neurol. 2022 Nov 3;13:1036798. doi: 10.3389/fneur.2022.1036798 (PMC9669069; doi:10.3389/fneur.2022.1036798)
Supplement: Supplementary file 1 [file Data_Sheet_1.DOCX]

**Appendix 1: Composite Autonomic Symptom Score (COMPASS-31) score**

1. In the past year, have you ever felt faint, dizzy, “goofy”, or had difficulty thinking soon after standing up from a sitting or lying position?

1) Yes

2) No (if you Marked No, please skip to question 5)

2. When standing up, how frequently do you get these feelings or symptoms?

1) Rarely

2) Occasionally

3) Frequently

4) Almost always

3. How would you rate the severity of these feelings or symptoms?

1) Mild

2) Moderate

3) Severe

4. In the past year, have these feelings or symptoms that you have experienced:

1) Gotten much worse

2) Gotten somewhat worse

3) Stayed about the same

4) Gotten somewhat better

5) Gotten much better

6) Completely gone

5. In the past year, have you ever noticed color changes in your skin, such as red, white, or purple?

1) Yes

2) No (if you marked No, please skip to question 8)

6. What parts of your body are affected by these color changes? (Check all that apply)

1) Hands

2) Feet

7. Are these changes in your skin color:

1) Getting much worse

2) Getting somewhat worse

3) Staying about the same

4) Getting somewhat better

5) Getting much better

6) Completely gone

8. In the past 5 years, what changes, if any, have occurred in your general body sweating?

1) I sweat much more than I used to
2) I sweat somewhat more than I used to
3) I haven’t noticed any changes in my sweating

4) I sweat somewhat less than I used to

5) I sweat much less than I used to

9. Do your eyes feel excessively dry?

1) Yes

2) No

10. Does your mouth feel excessively dry?

1) Yes

2) No
11. For the symptom of dry eyes or dry mouth that you have had for the longest period of time, is this symptom:

1) I have not had any of these symptoms

2) Getting much worse

3) Getting somewhat worse

4) Staying about the same

5) Getting somewhat better

6) Getting much better

7) Completely gone

12. In the past year, have you noticed any changes in how quickly you get full when eating a meal?

1) I get full a lot more quickly now than I used to
2) I get full more quickly now than I used to
3) I haven’t noticed any change

4) I get full less quickly now than I used to

5) I get full a lot less quickly now than I used to
13. In the past year, have you felt excessively full or persistently full (bloated feeling) after a meal?

1) Never

2) Sometimes
3) A lot of the time

14. In the past year, have you vomited after a meal?

1) Never

2) Sometimes

3) A lot of the time
15. In the past year, have you had a cramping or colicky abdominal pain?

1) Never

2) Sometimes
3) A lot of the time

16. In the past year, have you had any bouts of diarrhea

1) Yes

2) No (if you marked No, please skip to question 20)

17. How frequently does this occur?

1)  Rarely

2)  Occasionally

3)  Frequently ____________ times per month

4)  Constantly

18. How severe are these bouts of diarrhea?

1) Mild

2) Moderate

3) Severe

19. Are your bouts of diarrhea getting:

1)  Much worse

2)  Somewhat worse

3)  Staying the same

4)  Somewhat better

5)  Much better

6)  Completely gone

20. In the past year, have you been constipated?

1) Yes

2) No (if you marked No, please skip to question 24)

21. How frequently are you constipated?

1)  Rarely

2)  Occasionally

3)  Frequently ____________ times per month

4)  Constantly

22. How severe are these episodes of constipation?

1) Mild

2) Moderate

3) Severe
23. Is your constipation getting:

1)  Much worse

2)  Somewhat worse

3)  Staying the same

4)  Somewhat better

5)  Much better

6)  Completely gone

24. In the past year, have you ever lost control of your bladder function?

1)  Never

2)  Occasionally

3)  Frequently ____________ times per month

4)  Constantly

25. In the past year, have you had difficulty passing urine?

1)  Never

2)  Occasionally

3)  Frequently ____________ times per month

4)  Constantly

26. In the past year, have you had trouble completely emptying your bladder?

1)  Never

2)  Occasionally

3)  Frequently ____________ times per month

4)  Constantly

27. In the past year, without sunglasses or tinted glasses, has bright light bothered your eyes?

1)  Never (If you marked Never, please skip to question 29)

2)  Occasionally

3)  Frequently ____________ times per month

4)  Constantly

28. How severe is this sensitivity to bright light?

1) Mild

2) Moderate

3) Severe

29. In the past year, have you had trouble focusing your eyes?

1) Never (if you marked Never, please skip to question 31)

2) Occasionally

3) Frequently
4) Constantly

30. How severe is this focusing problem?

1) Mild

2) Moderate

3) Severe

31. Is the most troublesome symptom with your eyes (i.e. sensitivity to bright light or trouble focusing) getting:

1) I have not had any of these symptoms

2) Much worse

3) Somewhat worse

4) Staying about the same

5) Somewhat better

6) Much better

7) Completely gone
